# Using X-ray free electron lasers to explore extreme states under quasi-static compression

**DOI:** 10.1038/s41467-026-77171-2

**Published:** 2026-09-01

**Authors:** Rachel J. Husband, R. Stewart McWilliams, Mungo Frost, Zuzana Konôpková

**Affiliations:** 1 Deutsches Elektronen-Synchrotron DESY, Notkestr. 85, 22607 Hamburg, Germany; 2 Centre for Science at Extreme Conditions and School of Physics and Astronomy, University of Edinburgh, Edinburgh, UK; 3 SLAC National Accelerator Laboratory, Menlo Park, CA USA; 4 Present Address: European XFEL, Schenefeld, Germany

**Keywords:** Techniques and instrumentation, Planetary science, Structure of solids and liquids

## Abstract

The exceptional brilliance of X-ray free electron lasers (XFELs) combined with quasi-static compression in diamond anvil cells (DACs) has enabled novel time-resolved methods for probing extreme states of matter. Coupling serial X-ray techniques with dynamic drivers for rapidly changing pressure and temperature, these studies offer insight into phase equilibria, chemical reaction kinetics, and geophysical processes. Here, we present the current state-of-the-art XFEL techniques used for DAC research, including serial X-ray heating, pulsed laser heating, and rapid compression. Essential questions for future investigation are also highlighted, regarding the interactions of intense X-rays with samples at high pressure and associated non-equilibrium states.

## Abstract (web summary)

X-ray free electron laser pulses can create and probe extreme states of matter at timescales, conditions, and thermodynamic pathways which are inaccessible using conventional techniques. This perspective outlines notable results and questions raised by recent experiments on pre-compressed samples, and how these can be addressed in the future.

## Introduction

Our understanding of material behavior at high pressures (*P*) and temperatures (*T*) has been revolutionized by the use of ultrashort (<50 fs), intense (~1 mJ) pulses produced by X-ray free electron lasers (XFELs) to create^1^ and probe^2,3^ extreme *P–T* states. One of the primary tools for high *P* generation is the diamond anvil cell (DAC)^4^, comprising two opposing diamond anvils which can quasi-statically compress microscopic samples to *P* > 1000 GPa^5^. These devices are typically coupled with focused, brilliant X-ray beams for measurements of material properties at high *P* and *T* (up to ~10,000 K^6^) using diagnostics such as X-ray diffraction (XRD), spectroscopy, and imaging.

**Fig. 1:**
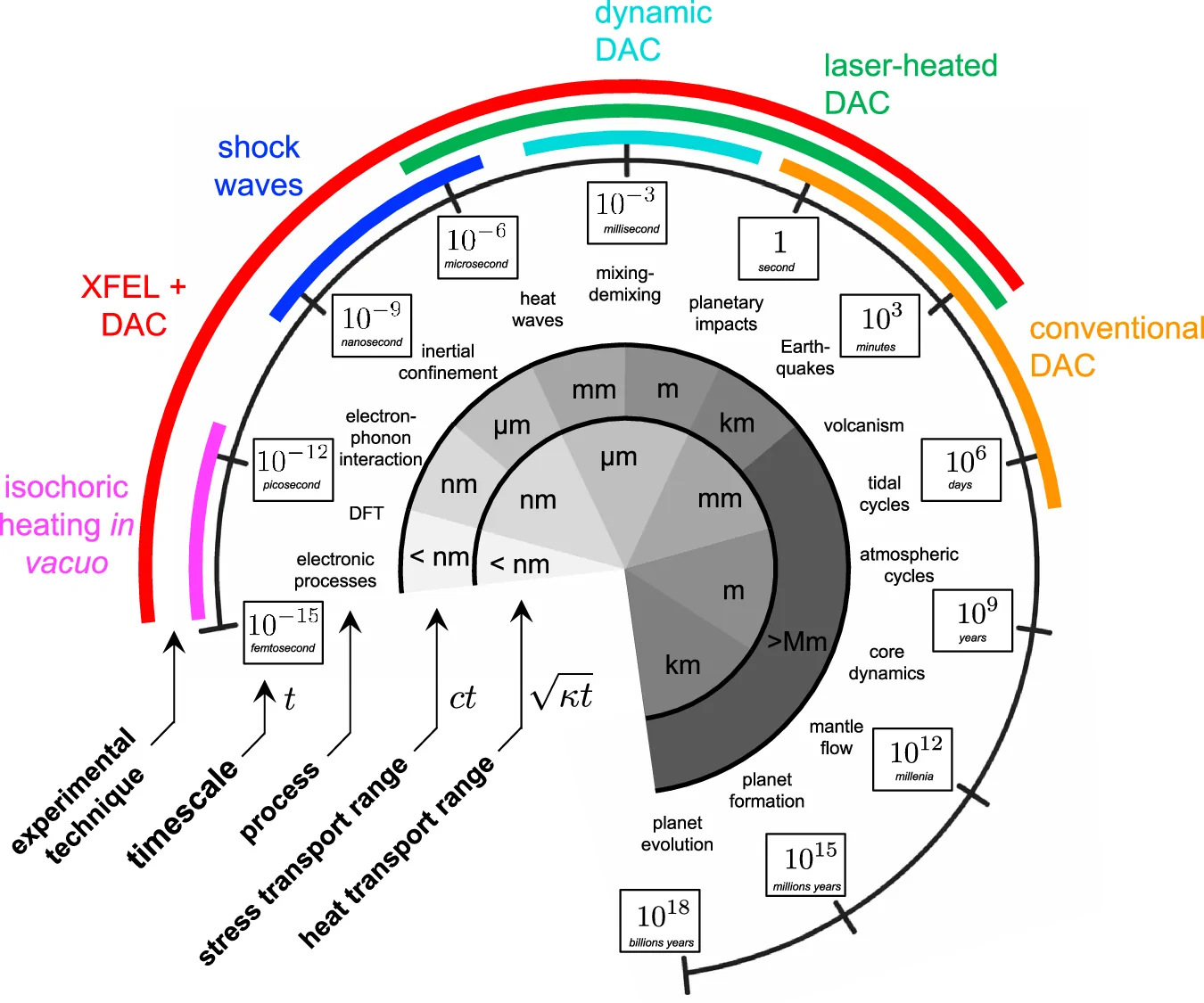
Timescales relevant to high pressure (*P*)–﻿temperature (*T*) processes and those accessible using common experimental techniques. X-ray free electron laser (XFEL) experiments in the diamond anvil cell (DAC) involve a uniquely wide range of timescales and, concomitantly, accessible physical processes. Representative length scales for *T* and stress transport are based on a representative sound speed of *c* = 1 km s^−1^ and thermal diffusivity of *κ* = 10^−6^ m^2^ s^−1^.

In recent years, time-resolved studies have benefited from advancements in pulsed sources and fast detection methods. In particular, DAC studies at XFELs became viable following the advent of hard XFELs^7^, which provide the high photon energies (>~12 keV) required to penetrate the diamond anvils. In these experiments, XFEL pulses are used not only as a fast structural probe to study dynamical processes induced by fast compression^8,9^ and IR laser heating^10,11^, but also as a pump to generate high *T* (>10,000 K)^12–14^ or induce photochemical reactions^15–17^. Anvil confinement suppresses the normally destructive processes induced by XFEL irradiation^18^, offering access to transient states^19^ and novel thermodynamic pathways^20^ which can be investigated in X-ray pump-probe experiments exploiting high-repetition rate XFEL modes^21^. Overall, these experiments offer insight into material response spanning an unprecedented range of timescales (Fig. 1) at extremes of *P* (>200 GPa)^22^, *T* (>10,000 K)^12^, and strain rate (10^3^ s^−1^)^8^.

This perspective discusses emerging state-of-the-art techniques used to study extreme states of matter in DACs with XFEL radiation, at conditions spanning hundreds of gigapascals and thousands of kelvin, as well as current scientific outcomes and future directions. From exploring the phase diagrams of planetary materials^22,23^, synthesizing exotic metal hydrides^17,24^, tracking chemical reaction kinetics^14,16^, and identifying short-lived metastable states during rapid compression^9^, the scope for DAC experiments at XFELs is being increasingly demonstrated. At the same time, previous experiments have raised many questions about the thermodynamic pathway and non-equilibrium physics activated by ultrashort X-ray irradiation, which are critically linked to the interpretation of experimental results, comparison with other experimental and theoretical techniques, and extrapolation to the long timescales of planetary interiors (Fig. 1). Understanding these processes will be greatly aided by future X-ray pump-probe experiments using shorter delay times^25,26^, which are currently available at XFELs but have not been employed in DAC research.

## Diamond anvil cell experiments at XFELs

The feasibility of XFEL DAC experiments was first demonstrated at PAL-XFEL (Pohang Accelerator Laboratory XFEL), where the structural response of samples irradiated with 12–14 keV X-rays was investigated using XRD^12,15^. Although direct probing of high *T* states was not possible due to the lack of in situ diagnostics, these experiments successfully demonstrated that focused XFEL pulses can induce melting^12^ and chemical reactivity^15^, and that DAC confinement remains robust under intense XFEL irradiation.

**Fig. 2:**
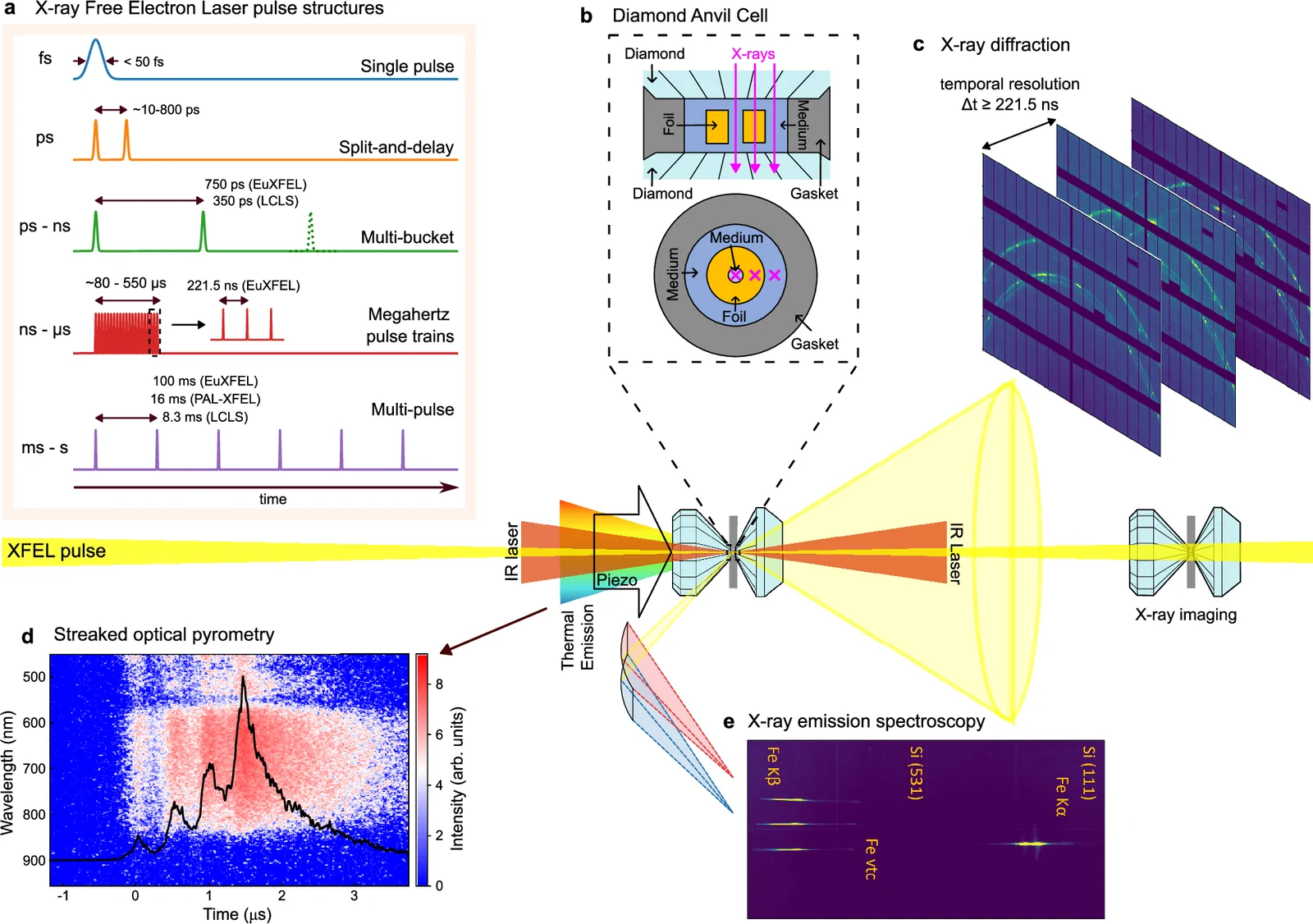
Layout of a typical diamond anvil cell experiment at an X-ray free-electron laser. **a** X-ray free electron laser (XFEL) pulse structures provided by the European XFEL (EuXFEL), the linear coherent light source (LCLS) and Pohang Accelerator Laboratory XFEL (PAL-XFEL), which can potentially be used to probe electronic relaxation (femtosecond to picosecond timescales), hydrodynamic release (picoseconds to nanoseconds), chemical kinetics and conductive cooling (microsecond) processes induced by hard XFEL irradiation^19^. Megahertz pulse trains can be coupled with IR heating lasers or piezoactuator-driven dynamic drivers^11,13^, and longer inter-pulse spacings (milliseconds to seconds) are well suited to XFEL-induced chemistry studies^15,40^. **b** Sample chamber of a diamond anvil cell containing a foil insulated from the diamonds by a surrounding medium. Different regions can be targeted by the focused XFEL beam for direct X-ray heating of the foil or medium, or indirect heating of the medium using the foil as an X-ray coupler^12–14^. The response of the sample can be studied using **c** X-ray diffraction, **d** streaked optical pyrometry, or **e** X-ray emission spectroscopy, with the implementation of X-ray imaging planned for the future. The images in **c** were adapted from ref. ^22^ and the figure in **d** was taken from ref. ^11^. Both are used under a Creative Commons (CC BY) license (http://creativecommons.org/licenses/by/4.0/). The image in **e** was collected during EuXFEL experiment 2941.

The unique pulse structure of the European XFEL (EuXFEL)^21^ opened new opportunities in high *P* science due to the unparalleled temporal resolution of its high-repetition-rate pulse trains (up to 4.5 MHz), which are now commonly employed for serial X-ray pump-probe experiments in DACs. The high-energy density (HED)-HIBEF instrument hosts the only dedicated DAC platform at a FEL^8,20^, equipped with pulsed IR heating lasers^11^, dynamic DACs^8^, and streaked optical pyrometry (SOP) for *T* determination^11^ (Fig. 2). The primary diagnostic is XRD performed using a flat-panel 10 Hz detector^20^ or a pulse-resolved detector which can resolve up to 352 pulses at megahertz repetition rates^27^. While photon energies as high as 25 keV are possible, all published DAC studies employing pulse-resolved XRD used ~18 keV X-rays. Complementary X-ray emission spectroscopy (XES) experiments have been performed at lower photon energies (~13 keV) with 10 Hz data collection rates^28,29^.

A DAC setup has also been demonstrated at the Linac Coherent Light Source (LCLS) using the 120 Hz hard X-ray linac^30^, which offers similar photon energies to EuXFEL. The LCLS-II-HE upgrades to the LCLS-II XFEL will offer photon energies potentially up to 20 keV at a sustained 1 MHz repetition rate. This will enable high temporal resolution probing of samples over longer times than typically used at EuXFEL, as well as unprecedented total X-ray flux.

## X-ray interaction with matter

**Fig. 3:**
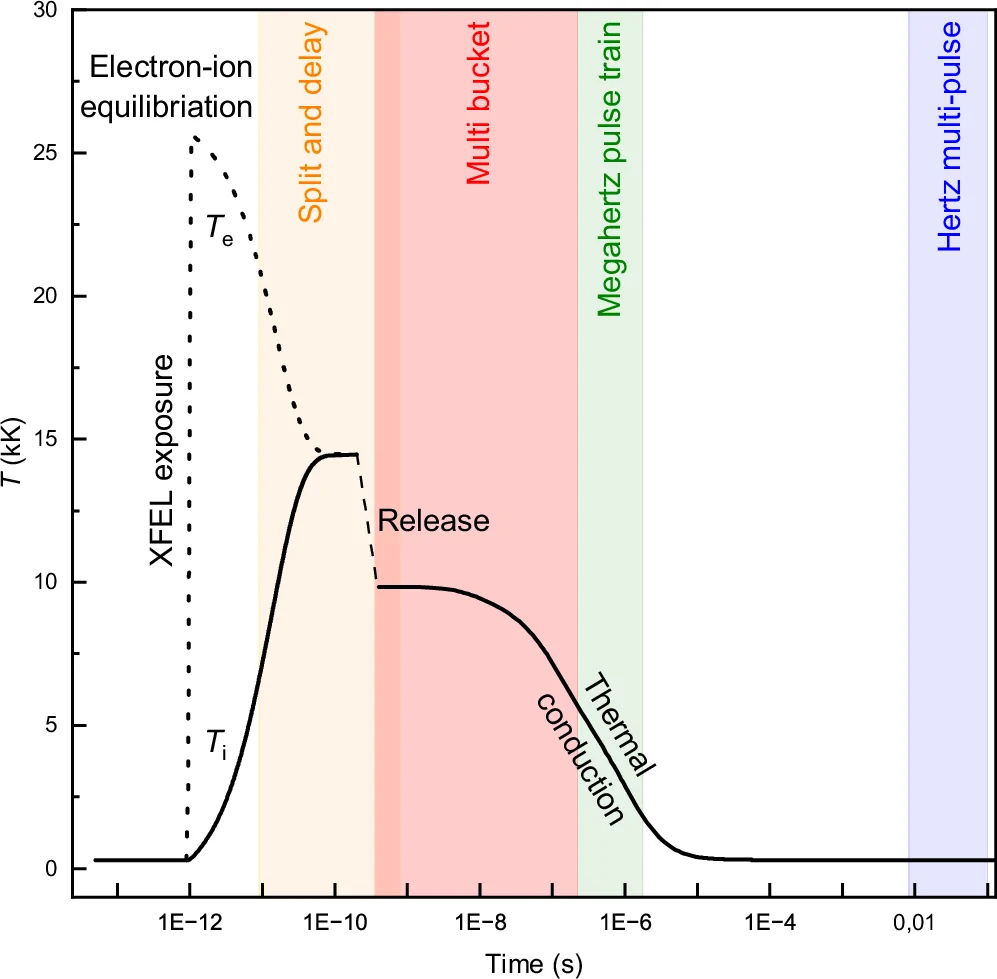
Simulated *T* evolution after irradiation of a compressed sample with a single X-ray free-electron laser pulse. An X-ray free electron laser pulse incident at 10^−12^ s produces a highly non-equilibrium state in which the temperature of the electrons (*T*_e_) is much higher than that of the ions (*T*_i_). The resultant electron–phonon thermalization, isentropic release, and thermal conductive processes occur across a broad range of timescales, which can be accessed in X-ray pump-probe experiments using different pulse structures^25,26,118,119,128^, as indicated by the shaded regions. The simulation was performed for an Au foil sample embedded in H_2_O (*P* = 4.7 GPa). Data are taken from ref. ^12^.

Ultrafast energy deposition caused by intense XFEL irradiation generates extreme *P–T* conditions that evolve over timescales ranging from femtoseconds to tens of microseconds (Fig. 3), inducing dramatic and potentially irreversible changes to the sample. XFEL pulse durations (<50 fs) are shorter than thermal melting^31^ and conventional thermal modifications to the lattice^32^, meaning that atomic positions are essentially fixed for the duration of the XFEL pulse. Consequently, scattering processes such as XRD provide a structural snapshot of the sample prior to energy deposition.

Hard X-rays primarily deliver energy to core electrons via photo-absorption, producing an initially non-thermalized electronic distribution which relaxes via fluorescence and Auger decay^33^, complex electron cascades^34^, and the transfer of energy from hot electrons to the lattice^35^. Lattice heating driven by electron-phonon coupling occurs on picoseconds timescales^32^, and can lead to thermal melting. Alternatively, the lattice can be destabilized by modifications to the interatomic potential driven by electronic excitation, resulting in ultrafast non-thermal melting within hundreds of femtoseconds^36,37^.

Heating on early timescales (<100 ps) is considered to be isochoric as thermal *P* has not yet been dissipated due to the finite sound speed of release waves. At later times, *P* is released into the surroundings via hydrodynamic expansion, potentially resulting in the destruction of the sample by a combination of melting, cavitation, and spallation^38^. Release and ablation can be delayed—or eliminated—by tamping samples with surrounding materials, which is the case in DACs^19^.

With destruction prohibited, *P* relaxes to equilibrium values within a few nanoseconds^19,38^, and the sample cools to room *T* via thermal conduction on microsecond to millisecond timescales^19^. Nominally, this later period can be considered in terms of conventional heat diffusion, as the *P–T* conditions do not strongly depend on the prior short-timescale evolution of the sample. Cooling times are heavily influenced by the high thermal conductivity of the diamond anvils (~2000 W/mK), which act as a heat sink.

**Fig. 4:**
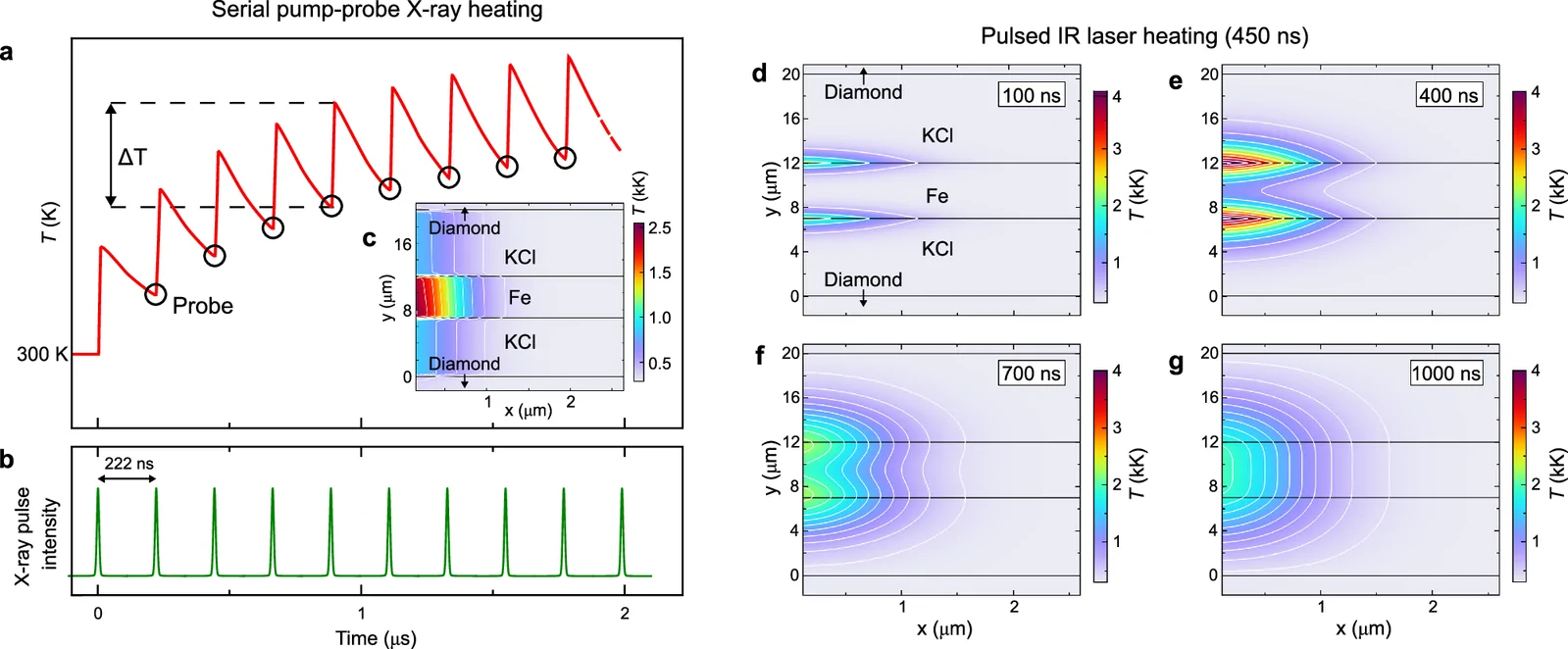
Temporal and spatial *T* profiles for different heating methods in diamond anvil cells. **a** A typical time-dependent *T* profile during irradiation with a 4.5 MHz pulse train from an X-ray free electron laser, where each pulse probes the sample as it cools from the state created by the previous pulse. This approach induces significant thermal oscillations (Δ*T*), which are not captured by the X-ray diffraction probe. The corresponding X-ray pulse structure is shown in **b**. **c** The spatial *T* distribution in an X-ray heated Fe sample in a diamond anvil cell, just after X-ray exposure, compared to **d**–**g** the spatial *T* distribution in the same Fe sample during pulsed IR heating (450 ns duration) at different delay times. Volumetric X-ray heating provides a relatively uniform *T* distribution within the irradiated region of the sample, whereas pulsed IR laser heating initially results in large axial *T* gradients because the laser heats the sample surface. The spatial *T* maps in **c**–**g** were determined from finite element analysis calculations using parameters similar to those used in ref. ^46^.

The high penetration depth of high-energy X-rays produces volumetric heating by depositing energy in the bulk of the sample (Fig. 4). X-ray absorption is heavily dependent on atomic number (~*Z*^4^), meaning that high *T* can be generated in high-*Z* materials. Typical DAC experiments can reach >10,000 K^12^, and much higher *T* (~100 eV) has been demonstrated on freestanding foils^39^.

There are two nominal limits for XFEL DAC experiments: non-perturbative (or minimally perturbative) probing using a low X-ray fluence to minimize heating and/or sample damage when using additional drivers^8–11^, and higher fluence experiments for novel applications including X-ray-induced materials synthesis^14–16,24,40^ and pump-probe X-ray heating^13,19,20^.

## State-of-the-art drivers and diagnostics

This section describes unique experimental approaches that have been employed in DAC studies at EuXFEL, highlighting their advantages over traditional techniques. Noteworthy results are described later in the text.

### X-ray heating

X-ray pump-probe experiments, where the first pulse heats the sample via fast energy deposition and the second probes the resultant high *T* state, are commonly employed for serial X-ray heating DAC experiments using megahertz pulse trains at EuXFEL. With pulse separations of 221.5 ns (and multiples thereof), samples are probed as they cool via thermal conduction (Fig. 3) at times significantly later than electronic and hydrodynamic relaxation processes, providing access to timescales, conditions, and thermodynamic pathways that were previously challenging to achieve.

Irradiation with a series of pulses initially produces a stepwise *T* increase (Fig. 4), which saturates when X-ray heating becomes equal to the heat dissipated between subsequent pulses^19^. Pulse-resolved XRD probes the sample’s structural response during heating for ~100 μs (the duration of the pulse train), facilitating thermal transport^41^ and chemical reactivity studies^14^. The pulsed nature of the XFEL source also produces pronounced thermal oscillations, potentially providing access to unique thermodynamic pathways^13,22^. Direct X-ray heating is possible even for samples that cannot be heated using IR lasers. Less absorbing low-*Z* samples can benefit from the use of an embedded high-*Z* coupler, which is directly heated by the XFEL beam and heats the low-*Z* material via thermal conduction^13,14^ (Fig. 2).

Short timescale heating offers several advantages over prolonged, continuous wave (CW) heating, including higher achievable *T* due to the reduced risk of diamond/gasket failure^6^ and the suppression of unwanted chemical reactions. In particular, contamination with carbon transported from the diamond anvils, which occurs where experimental timescales exceed those of diffusion^42^, is a well-known problem in LH-DAC studies and has been proposed to account for earlier discrepancies in melting curve measurements of Fe^43^.

The *T* evolution during X-ray heating can be estimated using SOP^11^, where the time-resolved thermal emission spectrum is fit to a Planck function to determine a *T* and relative emissivity. *T* can be further constrained by finite element analysis (FEA) modeling based on the known sample geometry and material properties, and the measured X-ray energy delivered to the sample^10,13,14,19,22^. FEA models predict very high transient *T* in high-*Z* materials^19^, such as the metallic couplers used to heat lower *Z* samples^13^, though oscillations are damped further from the coupler^14^.

### Pulsed IR laser heating

The laser-heated DAC (LH-DAC) is a standard technique employing focused, near-infrared lasers to generate high *T* in statically compressed samples, serving as a complementary method to serial XFEL heating. The laser heating system at the HED-HIBEF instrument^11^ has enabled XFEL probing of material behavior during heating with a single, short-timescale (20–420 ns) IR laser pulse^10,11^, maintaining the short-timescale heating advantages described above. Longer pulse durations (several hundreds of microseconds), which are suitable for melting and recrystallization experiments using the EuXFEL pulse train, are also possible. Collecting high-quality XRD data in single-shot, high *T* (> 10,000 K) experiments is crucial for measurements of incongruent (i.e., partial) melting, as chemical migration driven by *T* gradients can alter the composition of the probed area during prolonged heating^44^. For example, a 250 ns pulse was shown to be sufficiently short to suppress chemical migration in Fe–Si–O alloys, allowing for the liquidus *T* to be determined based on recrystallization from the melt^10^.

Although optical and X-ray heating share some basic similarities, there are also considerable differences. Serial X-ray heating is time-varying due to the intrinsically pulsed nature of the source, and the detailed *T* distribution differs due to volumetric heating using X-rays^10,19^ vs. surface heating^45,46^ using IR lasers (Fig. 4). Although pulsed optical heating can produce very high peak *T* on the sample surface, XRD measurements are challenging due to the presence of large axial *T* gradients. Another crucial difference pertains to laser coupling behavior; optical heating depends on detailed geometries (i.e., surface finishes and optical cavity interferences) and the sample’s optical properties and their dependence on *T* and phase (e.g., solid vs melt). X-ray heating is not susceptible to these constraints, depending only on the X-ray absorption properties of the sample assembly.

### Dynamic compression at intermediate strain rates

Material behavior at high strain rates ($\dot{\epsilon }$) is of interest to a diverse range of scientific disciplines, ranging from asteroid impacts relevant to Earth and Planetary science to crash events studied by the aerospace and automotive industries. Experiments are typically performed using shock waves ($\dot{\epsilon } > 1{0}^{5}$ s^−1^), which can generate the ultra-high *P–T* conditions found in planetary interiors. Intermediate strain rates ($\dot{\epsilon } \sim 1{0}^{-1}-1{0}^{3}$ s^−1^), which cannot be accessed by traditional static and shock compression techniques, have also opened new avenues for scientific exploration: metastable phases formed at modest (de)compression rates^9,47,48^ offer opportunities for material synthesis; simulating asteroid impacts can identify mechanisms responsible for shock-metamorphism^49^; and elastic softening determined from *P* oscillations can provide insight into seismic wave velocities in mantle minerals^50^.

Intermediate strain rates can be generated by dynamic DACs (dDACs) employing piezoelectric actuators for rapid *P* control^51,52^. Although experiments above $\dot{\epsilon } \sim 1{0}^{-1}$ s^−1^ are scarce due to the lack of time-resolved diagnostics, the recent implementation of dDACs at the HED-HIBEF instrument has extended measurements up to $\dot{\epsilon } \sim 1{0}^{3}$ s^−1^^8^, where the structural response of the sample can be studied with MHz XRD performed using a single pulse train (~550* μ*s in the ‘long train’ mode) with microsecond temporal resolution^53^.

**Fig. 5:**
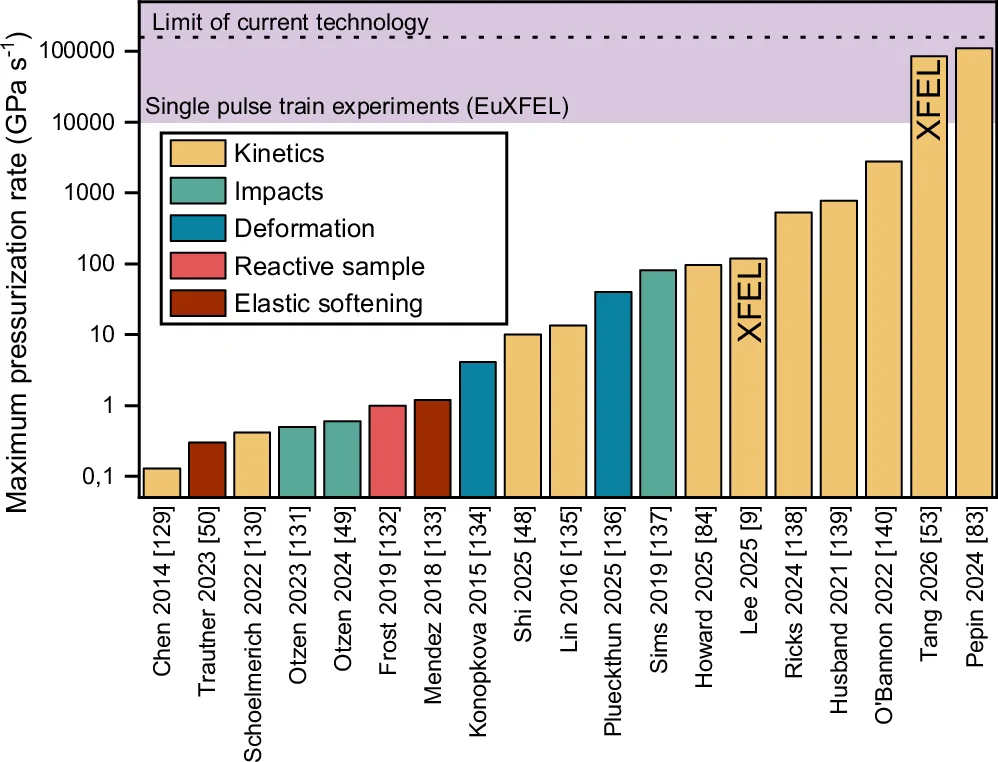
Fast compression using dynamic diamond anvil cells. Maximum pressurization rates in previous studies which used X-ray probes for structural determination^9,48–50,53,83,84,129–140^. All works not labeled `XFEL' were performed at a synchrotron source. Colors indicate the scientific focus of the work. Pressurization rates suited to probing with a single pulse train at the European X-ray free electron laser (EuXFEL, purple shaded region) remain relatively unexplored. To date, studies relevant to Earth and planetary science (impacts, deformation, and elastic softening) have been limited to below ~100 GPa s^−1^, well below the limit of current technology (~160 TPa s^−1^), and would greatly benefit from experiments at X-ray free electron laser sources.

Early XFEL dDAC studies focused on prototypical phase-transforming materials to demonstrate platform capabilities^8^, and extension to a wider range of sample types promises new opportunities for scientific discovery. In particular, Synchrotron studies relevant to Earth and planetary science have been limited to below ~100 GPa s^−1^ (Fig. 5), which are significantly lower than those experienced in large-scale impacts. Faster experiments at XFELs would therefore be of substantial interest to the community.

## Outstanding results

### Planetary science

In recent years, serial X-ray heating in DACs has been used to investigate material behavior at planetary interior conditions, offering distinct advantages over traditional methods such as LH-DACs. In addition to the benefits offered by volumetric, short-timescale heating, high data collection rates have enabled the determination of material properties based on dynamical material response^41^ and the detection of short-lived, intermediate states^9^.

Geological modeling requires accurate knowledge of transport properties such as thermal conductivity, which is challenging to measure at high *P–T* conditions. The thermal conductivity of bridgmanite—the most abundant mineral in Earth’s lower mantle—was measured at lower mantle conditions (up to 60 GPa and 3100 K) in serial X-ray heating experiments^41^. The thermal conductivity was extracted from FEA models based on the known sample geometry, energy deposition, and the temporal evolution of *T* experimentally constrained by the thermal equation of state (EoS) of the sample and by SOP. While a range of high *P* thermal conductivity measurement techniques exist^54^, the X-ray heating approach has the advantage that it is compatible with simpler (ideally single-component) sample geometries, which greatly simplify thermal transport modeling. The results on bridgmanite set new constraints that are directly measured and not reliant on scaling laws, helping to refine the heat flux at the core-mantle boundary.

**Fig. 6:**
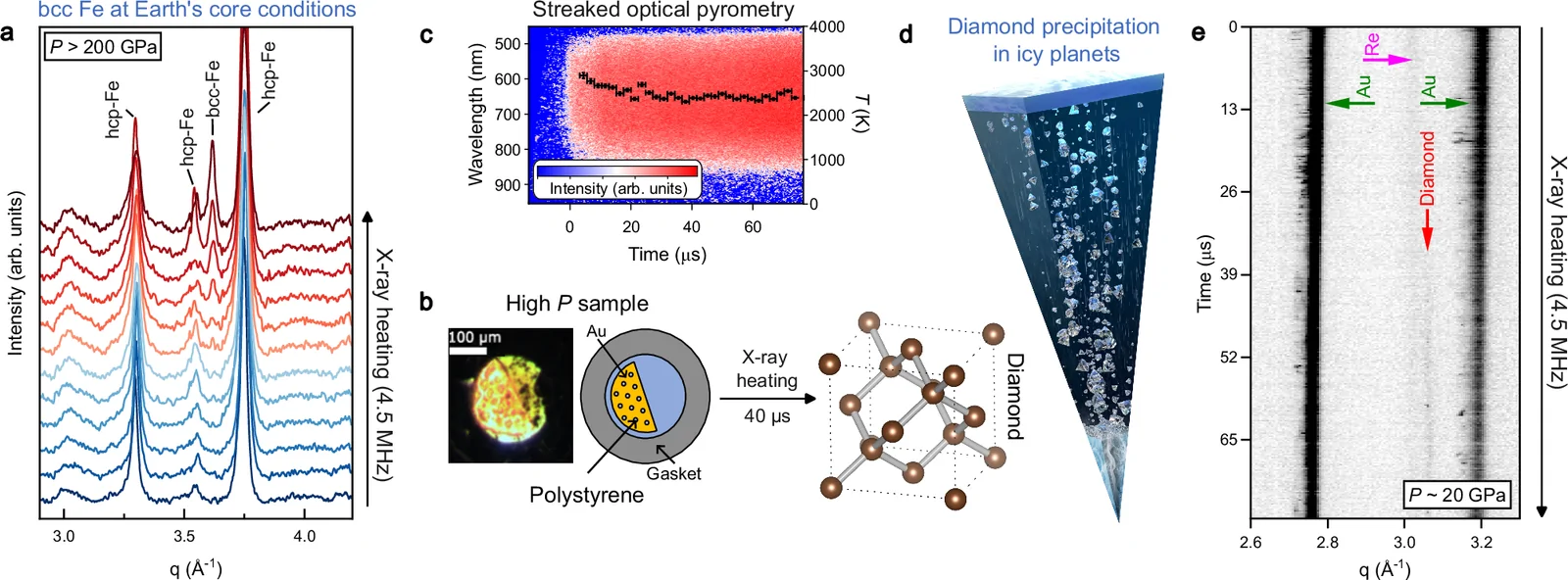
Exploration of planetary interiors using X-ray heated diamond anvil cells. **a** Integrated X-ray diffraction pattern showing the formation of bcc-Fe at Earth’s core conditions (>200 GPa) during serial X-ray heating. Data are taken from ref. ^22^. **b** Photomicrograph and illustration of a polystyrene sample in the sample chamber of a diamond anvil cell, which formed diamond when indirectly heated above 2000 K for ~40 μs using an embedded Au X-ray coupler. **c** Corresponding streaked optical pyrometry spectrogram and calculated *T* evolution for data shown in **a**. **d** The formation of diamond at ~20 GPa suggests that diamond precipitation from hydrocarbons occurs at deeper depths in icy solar planets than previously thought. **e** Diamond formation was identified from pulse-resolved X-ray diffraction. Data are taken from ref. ^14^.

Moving deeper into the planet, the stable structure and properties of Fe in the core are important for understanding the thermal structure and magnetic field of the Earth. Until recently, scientists broadly agreed that Fe in the core is hcp; however, hcp-Fe is close in energy to various cubic phases at these conditions^55^. Studying phase transitions in Fe is challenging, as dynamic compression can potentially access metastable phases due to slow transition kinetics^56^, while longer heating timescales can present issues with contamination^57^. Serial XFEL heating experiments observed the formation of bcc-Fe above 200 GPa and 4400 K^22^ (Fig. 6). Total heating durations of ~77 μs were orders of magnitude longer than dynamic compression experiments, but fast enough to limit material diffusion and prevent contamination. However, the authors raised the question of whether the observation of bcc-Fe was related to the fast heating/cooling cycles in these experiments, with bcc-Fe formation being stabilized by the solidification process. The ability to access high-*P* metastable states via rapid thermal cycling is a unique capability of XFEL-heated DACs, but poses the challenge of distinguishing metastable structures from those that are thermodynamically stable. Identifying path-dependence requires precise knowledge of the time-dependent *T* profile in the experiment, which currently relies heavily on theoretical models. Future X-ray pump-probe experiments at short time delays may offer further insight, while improvements to pulsed LH-DACs at XFELs will offer the opportunity to compare different heating pathways.

XFEL methods have also been used to investigate diamond precipitation in the interiors of icy planets such as Uranus and Neptune. Prior studies reported very different thresholds for diamond formation from hydrocarbons depending on the compression technique: diamond formation from methane occurred in LH-DAC experiments above 10 GPa and 2000–3000 K^58,59^, whereas diamond formation from polystyrene was not observed until above 140 GPa and 5000 K during laser-driven shock compression^60^. Serial XFEL heating experiments on polystyrene observed diamond precipitation at 20 GPa and 2200 K, but on 30 μs timescales^14^ (Fig. 6), revealing the origin of the discrepancy between previous studies to be experimental duration. The lower *P* onset of diamond precipitation places it at shallower depths in Uranus and Neptune—above the superionic (SI) ice layer—such that dense diamond sinks further when it falls into the planet under gravity, acting as a heat source for a larger region of the planet. Subduction plumes of diamond-rich material may also affect convection in SI ice, potentially contributing to the planets’ complex magnetic fields^61^.

Despite numerous XFEL DAC experiments probing planetary interior conditions, there remain key questions that must be addressed before results are used to determine the stable phases, physical properties, and chemical processes inside planets. The possibility that metastable phases form during fast cooling^62^ may explain observed differences compared to other experimental approaches^13,22^, but the thermodynamic pathway induced by XFEL heating and the nature of spatiotemporal *P–T* gradients are not well understood^23^. The physics induced by X-ray-electron interaction and its influence on phase transitions and chemical processes is also an open question, which will be further discussed in the following section.

### XFEL-induced chemistry

The chemical behavior of many systems is modified by *P*, leading to the formation of exotic compounds with complex chemical bonding^63,64^ and stoichiometries^65–67^. Novel chemistry can also be induced by hard X-ray irradiation, which triggers electronic relaxation processes that can potentially access new reaction pathways and synthesis conditions. Hard synchrotron radiation has been employed in DAC studies to initiate decomposition in a range of molecular compounds^68–72^, and XFEL irradiation of compressed samples can provide access to new, potentially metastable, states either due to the high *T* arising from X-ray heating or from direct photochemical effects.

**Fig. 7:**
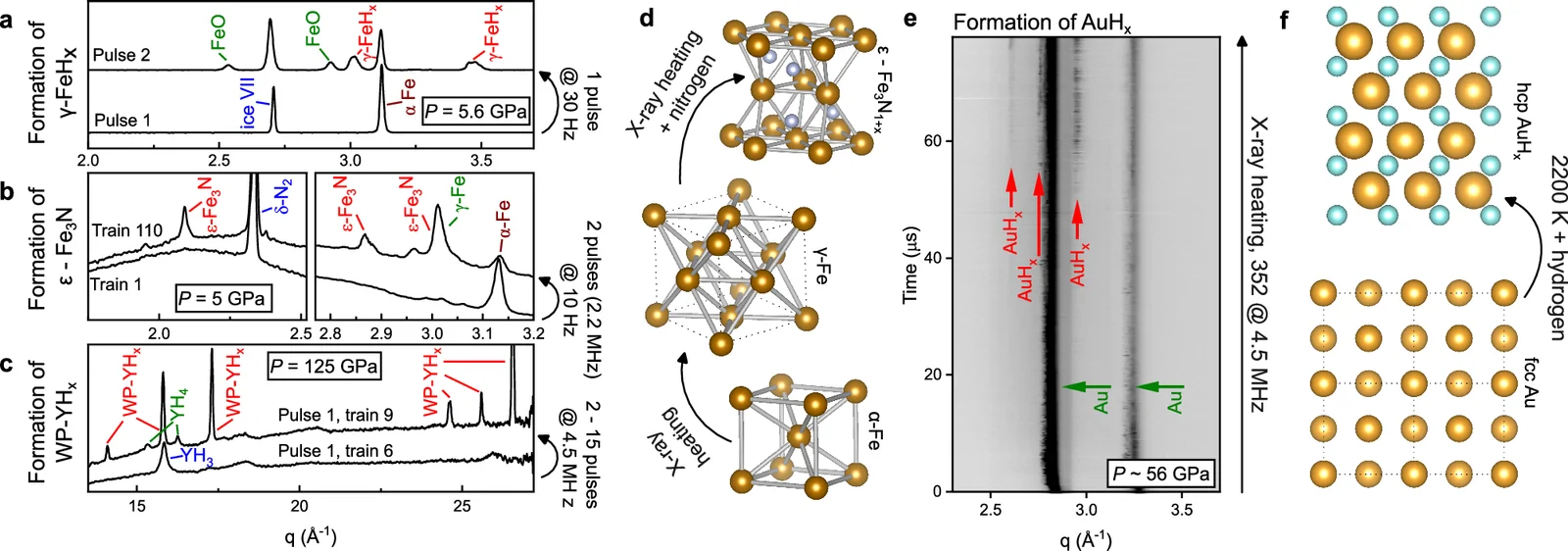
X-ray free-electron laser-induced chemistry in diamond anvil cells. Integrated X-ray diffraction patterns illustrate the formation of **a** *γ*-FeH_*x*_, **b** *ϵ*-Fe_3_N, and **c** WP-YH_*x*_ induced by different irradiation conditions. In **a**, XRD probed the room *T* state due to the low repetition rate (30 Hz) of the source. The 10 Hz data collection rate in **b** meant that signals from pump and probe pulses (2.2 MHz) were contained within a single pattern, whereas **c** used pulse-resolved detectors. The reaction in **b** is illustrated in **d**. **e** Pulse-resolved X-ray diffraction patterns showing the formation of AuH_*x*_, as illustrated in **f**, which is only stable at high *T*. Data in **a**–**c** and **e** are taken from refs. ^16, 17, 24, 40^, respectively.

To date, XFEL-induced chemistry studies using DACs have focused on the synthesis of binary nitrides and hydrides. A pioneering study at PAL-XFEL^15^ observed the rapid formation of H_2_S in sulfur loaded in H_2_ (*P* = 0.3 GPa) after irradiation with 12 keV X-ray pulses with a delay between exposures of seconds or more. Subsequent work at EuXFEL^16^ exposed Fe in N_2_ (*P* = 5 GPa) to a series of pulses at 2.25 MHz and 17.8 keV, resulting in the formation of *ϵ*-Fe_3_N_1+*x*_ (*x* = 0.33) (Fig. 7). Although H_2_S and *ϵ*-Fe_3_N_1.33_ were already known, these early studies demonstrated the ability of XFEL irradiation to access new synthesis pathways.

XFEL-induced synthesis of metal hydrides of Fe^40^, Y^17^, and Au^24^ has also been reported (Fig. 7). A previously unobserved Au hydride (Au_2_H_*x*_ with *x* < 1) forms above 40 GPa and 2200 K during XFEL irradiation at 4.5 MHz (18 keV), reverting back to fcc Au with undetectable dissolved hydrogen on cooling to 295 K. Its instability on quenching suggests that high *T* is required for its stability. In contrast, the synthesis of a previously-unobserved Y hydride (WP-YH_*x*_ with 4 ≤ *x *≤ 5.7)^17^ was attributed to non-equilibrium pathways accessed by intense X-ray irradiation, based on the lack of thermal emission in SOP implying a maximum *T* below 1500 K^11^.

The transient conditions generated by XFEL irradiation have also been used to simulate chemical reactions in giant impacts, between the metallic core and the major volatiles of the proto-Earth^40^. Irradiating samples of Fe in H_2_O or CO_2_ with 30 Hz XFEL pulses (14 keV) led to the oxidation of Fe into wüstite (FeO), which, depending on the *P* and volatile type, was potentially followed by the formation of Fe hydride (*γ*-FeH_*x*_) or siderite (FeCO_3_). The type and extent of formation of reaction products, and their dependence on *P*, have implications for mantle chemistry in the early Earth.

Ascertaining the role of intense X-ray exposure in XFEL-induced chemical processes, which can be photochemical or thermal in nature, poses a significant challenge, especially when results are to be applied to planetary interiors. For example, the recent observation of solid methane^73^ at conditions where diamond forms from compressed polystyrene during XFEL irradiation^14^ raises the question of whether diamond formation in these experiments proceeded via photochemical dissociation. Low pulse repetition rates, for example, 30 Hz at PAL-XFEL or 120 Hz at LCLS, allow samples to cool between the pulses, greatly reducing thermal buildup, and can be used to determine whether a reaction observed in serial XFEL-heated samples occurs in the absence of sustained high *T*^30^. Advanced XFEL pulse modes, which can probe intermediate states femtoseconds to picoseconds after irradiation, can potentially shed light on photochemical processes, as well as open new avenues for X-ray femtochemistry in DACs.

### Water science

Knowledge of water’s properties under extreme *P* and *T*—up to hundreds of gigapascals and thousands of Kelvin—is crucial for planetary modeling due to the vast quantities thought to be present in the interiors of the outermost Solar planets^74^ and water-rich exoplanets. In particular, water has been proposed to contribute to Uranus and Neptune’s unusual magnetic fields, which have been explained by a thin shell of ionized water surrounding a deep superionic (SI) ice layer^75^, or a conductive H_3_O layer near the core^76^. Consequently, SI ice, characterized by a high ionic conductivity resulting from highly mobile protons, has been the focus of numerous DAC studies^77–79^, which have not yet reached a consensus on the stability fields of SI-bcc and its close-packed structures (SI-fcc and SI-hcp^80^).

**Fig. 8:**
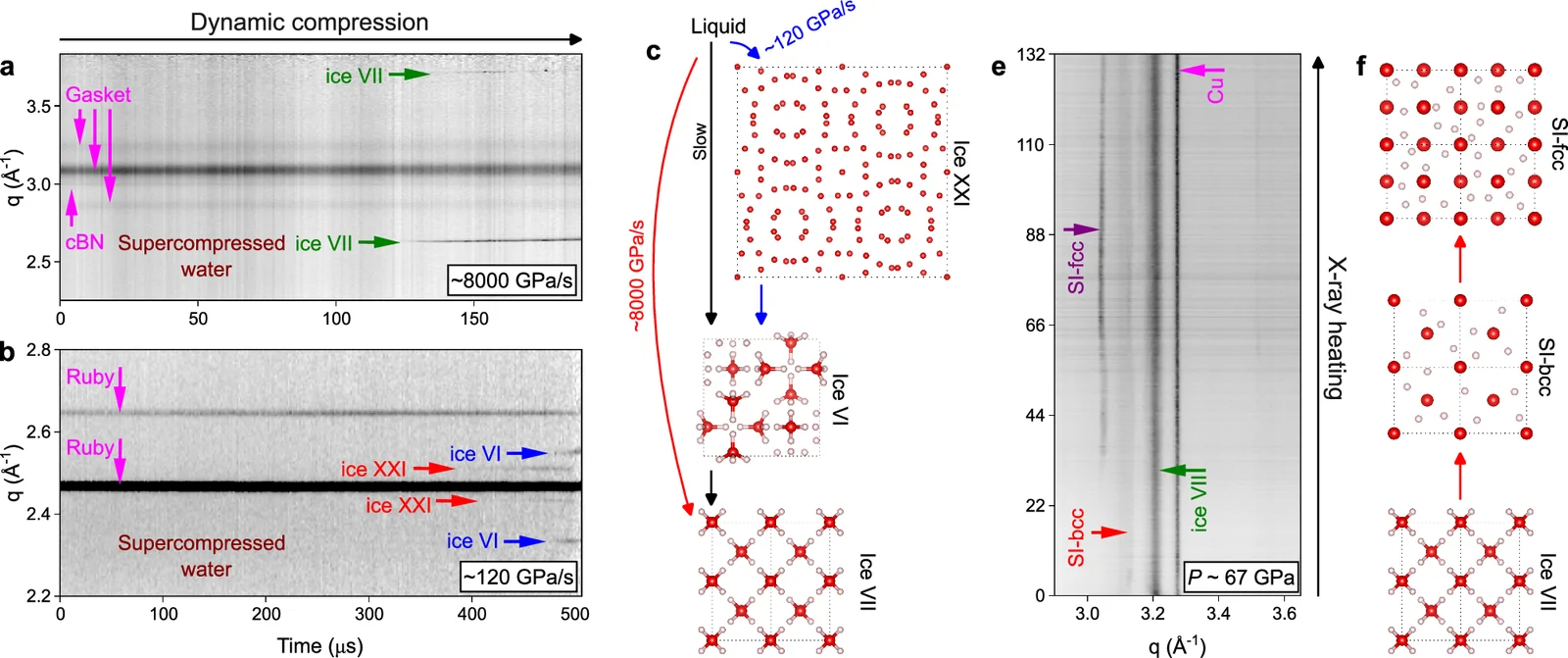
Exotic phases of H_2_O created in X-ray free-electron laser experiments. Integrated X-ray diffraction patterns illustrating different thermodynamic pathways which can be accessed during the dynamic compression of H_2_O at **a** ~8000 GPa s^−1^ and **b** ~120 GPa s^−1^. Supercompressed water transforms directly to ice VII in **a**, bypassing the equilibrium ice VI phase, whereas crystallization proceeds via the metastable ice XXI phase in **b**. Crystal structures are illustrated in **c**. **e** Integrated X-ray diffraction patterns showing the formation of superionic ice phases (SI-bcc and SI-fcc) during X-ray heating at ~67 GPa. Structural changes are illustrated in **f**. Data in **a**, **b**, and **e** are taken from refs. ^8, 9, 13^, respectively.

Using XFELs to heat H_2_O limits the likelihood of unwanted chemical reactions, both by access to inert X-ray couplers which cannot be heated by IR lasers, and the short heating durations made possible by high-quality XRD obtainable from a single XFEL pulse. The use of an embedded Au foil to indirectly heat H_2_O was first demonstrated at PAL-XFEL^12^, where a porous structure in the recovered Au provided evidence of vigorous mixing of molten Au and H_2_O, and XRD showed no evidence of previously-reported X-ray induced dissociation products^68^. The EoS and stability fields of SI ice were subsequently investigated in serial X-ray heating experiments at EuXFEL^13^, which employed a range of coupler materials to create and probe SI ice using 2.25 MHz XFEL irradiation at 18 keV (Fig. 8). The success of this experiment relied on two unique aspects: (1) the SI ice signal used for EoS determination was enhanced by a new data analysis methodology, which identified diffraction from individual crystallites across the entire XRD dataset, and (2) the *T* profile induced by serial XFEL irradiation revealed evidence of phase transition kinetics associated with SI-fcc formation from the melt. The preferential crystallization of SI-bcc may have implications for the interior dynamics of icy planets, where SI-bcc formed at solid/fluid boundaries in convection processes could influence physical properties such as thermal and electrical conductivity in ice layers.

Early dDAC studies employing spectroscopic diagnostics found that metastable phases are also formed in H_2_O during rapid compression at room *T*, with supercompressed water crystallizing in the ice VII structure in the stability field of ice VI^81^ before transforming to high-density amorphous (HDA) ice^82^. The absence of ice VI during rapid compression was confirmed by XRD experiments performed at EuXFEL^8^ (Fig. 8), and subsequently by synchrotron experiments^83,84^ in which liquid water was maintained up to ~2 GPa above its equilibrium stability field^83^.

More recently, an EuXFEL dDAC experiment revealed that the freezing/melting process is more complex than originally thought, identifying five distinct transition pathways within the stability field of ice VI^9^. In this work, the authors successfully captured the onset of crystallization in samples subjected to a series of 10 ms compression/1 s decompression cycles, revealing that crystallization sometimes occurs via the metastable ice XXI phase, the formation of which is directly linked to the structural evolution of supercompressed water (Fig. 8). The existence of metastable phases at room *T* motivates the extension of these experiments to higher *T*, where the metastability limit of liquid water has been extensively studied using isentropic compression techniques^85^.

## Current challenges and future outlook

Novel discoveries in XFEL DAC research are supported by continuous developments in sources, detectors, and experimental configurations. Here, we outline prospective X-ray methodologies and alternative *P–T* drivers that could be enabled by or benefit from XFEL capabilities, before discussing the key questions raised by previous XFEL DAC experiments and how these could be addressed in future experiments.

### X-ray diagnostics

*X-ray imaging* is routinely used in static high-pressure experiments at synchrotrons^86–89^, with partially coherent sources more recently enabling X-ray phase-contrast imaging (XPCI)^12,88^. These techniques are ideal for investigating melting^12,88,90^, viscosity^86^, liquid immiscibility^87^, and the density of amorphous materials^89^. Meanwhile, various imaging modalities, including bright- and dark-field^91^, XPCI^92^, and Talbot interferometry^93^, are increasingly employed to image ultrafast phenomena at XFELs, taking advantage of high coherence. In DAC experiments, MHz^94^ to GHz^95^ XFEL imaging will provide the temporal resolution necessary to probe dynamical processes, including melting and crystallization during rapid thermal changes, mixing and demixing phenomena, particle movements for viscosity determination, and wavevector-dependent dynamics via X-ray differential dynamic microscopy^96,97^. At the same time, ultrashort XFEL pulses will eliminate motion blurring caused by interface and particle movements while enabling studies on opaque matter where optical techniques are excluded.

*X-ray emission spectroscopy (XES)*, which provides element-specific information on local structure and electronic spin state, is well-suited for investigating spin transitions in Fe-bearing minerals, which play an important role in deep Earth geodynamics. XES was previously employed in XFEL heating DAC experiments (~13 keV) to identify spin-state changes FeCO_3_^28,29^; however, this work used 10 Hz data collection rates where the XES signal from each pulse train was contained within a single image. Future time-resolved studies using a megahertz detector^98^ will isolate the high *T* signal, and the combination of XES with SOP will provide simultaneous *T* determination, which was not possible in previous work.

*X-ray absorption spectroscopy (XAS)*, comprising X-ray absorption near edge spectroscopy (XANES, sensitive to oxidation state and coordination geometry) and extended X-ray absorption fine structure (EXAFS, sensitive to local structure), is standard in DAC Synchrotron studies but challenging at XFELs due to the limited bandwidth (10s of eV) and shot-to-shot spectral fluctuations. While single-shot XANES^99,100^ and multi-shot EXAFS are possible at XFELs^100^, DAC experiments can be complicated by the high absorption of diamond at the absorption edge (e.g., the Fe K-edge at ~7.1 keV), limiting X-ray heating and entering a regime where diamond anvil stability remains untested. Nevertheless, combining dispersive XANES with dynamically heated DACs will benefit from the short-timescale heating advantages to extend measurements on geomaterials^43,101,102^ to higher *T* and into time-domain observations. Key observations such as saturable absorption at hard X-ray energies^103^ also may be extendable to above solid density conditions with DAC techniques.

*Small-angle X-ray scattering (SAXS)* provides nanoscale (1–100 nm) information complementary to the atomic-level details provided by XRD. Performing time-resolved SAXS at XFELs will shed light on phase transition mechanisms, mixing/demixing transitions, and reveal growth rates of chemical reaction products. SAXS has previously been used to characterize diamonds formed from hydrocarbons during shock compression^104^ and in quenched LH-DACs^105^. In situ high *T* SAXS measurements will further characterize diamond formation kinetics, which was proposed to account for the discrepancy between studies with different experimental durations. The high coherence of XFEL beams also offers the possibility of high repetition-rate X-ray photon-correlation spectroscopy (XPCS) to probe internal dynamics such as relaxation times in glass-forming systems such as amorphous ice^106^.

### Pressure and temperature drivers

*Rapid compression experiments* combining dDACs with optical IR laser heating will extend measurements to high *T*^107^ accessing states more directly comparable to conventional high-strain-rate experiments conducted with shock methods, and enabling isothermal EoS measurements of reactive materials at planetary interior conditions. X-ray heating offers an alternative approach to achieve higher *T* in rapid compression, but raises the question of how fast *T* oscillations will influence phase transformation kinetics. Radial diffraction, in which the in the incident beam passes through the gasket (perpendicular to the applied stress axis) could also in principle be fielded at XFEL sources, for example to look at texture and deformation at different strain rates^108^.

*Pre-compression techniques*, in which DACs are coupled with shock compression to access Hugoniot states at higher initial density, often accessing conditions that are more relevant to planetary interiors, have been demonstrated using large-scale laser facilities^109^. Combining XFEL probing with these approaches can enable powerful characterization methods, and such experiments will benefit from sufficiently high-energy long-pulse drive lasers being co-located with XFEL facilities. Moreover, XFEL pumping offers a novel strategy for internally driven pre-compressed shock wave generation^19^.

*Short pulse lasers* with sub-picosecond pulse durations can be used to excite non-equilibrium states even with the limited intensity required to stay below the damage threshold of the diamond anvils^30^. Short pulse laser pump–XFEL probe experiments at picosecond delays have the ability to probe ultrafast phenomena and non-equilibrium dynamics such as electron-phonon coupling ^31^, molecular dissociation^110^, non-thermal phase transitions^36,37,111^, and melting dynamics of superheated solids^112^. Study of such phenomena has been focused on ambient density, but the development of XFEL DAC experiments will enable the effect of static pressure to be studied.

### Uncertainty in the *P–T* path

Serial X-ray heating is a powerful tool for creating and probing high *T* states in DACs. However, recent results have raised critical questions about the conditions induced by X-ray heating and physics activated by intense XFEL irradiation, which are not yet fully understood. For example, how can the effects of *T*, timescale, and X-ray-driven photochemistry be disentangled in chemical reaction studies? Are metastable, high *T* phases formed during heating or quenching from the melt? How does XFEL irradiation influence phase transition kinetics during dynamic compression? Furthermore, ultrafast phenomena driven by electronic excitations remain completely unexplored at static high *P*. Understanding these processes is critical for interpreting results from serial X-ray heating experiments, especially if results are used for modeling of long-timescale processes such as those in planetary interiors.

To date, X-ray pump-probe experiments have been limited to 4.5 MHz, meaning that knowledge of the sample behavior within the first 221.5 ns is heavily reliant on hydrodynamic^19^ and FEA models^10,13,14^ and comparison with untamped studies^38^. However, models are heavily dependent on material properties and X-ray beam parameters, which are not always well characterized. Although *T* can be experimentally constrained using XRD or SOP, both approaches have limitations. XRD can provide *T* bounds based on thermal expansion or melt detection (if a well-constrained high *T* EoS or melt curve is available), but data interpretation is complicated by smeared diffraction peaks resulting from large spatial *T* gradients^23^. Melt detection provides a lower *T* bound^24^, but diffuse scattering is not always observed. In SOP, thermal oscillations induced by individual pulses can be captured using short streak windows (1–5 μs), but measurements require signal accumulation due to reduced signal. This limitation will be addressed in future upgrades to the HED-HIBEF platform by integrating alternative pyrometry diagnostics, such as a multicolor system based on photomultiplier tubes. This will offer a higher temporal resolution and improve sensitivity at low *T*, which will improve our understanding of the thermodynamic pathways of XFEL-heated samples, though a full understanding of the state of the sample in the nanoseconds after XFEL exposure will ultimately require experiments using shorter X-ray delay times.

### New opportunities at XFEL sources

A central challenge for megahertz X-ray pump-probe experiments is the lack of direct access to ultrafast processes that occur between successive pulses (Fig. 2). Recent and forthcoming developments at XFELs promise to provide access to this temporal window, extending the scope of DAC experiments at XFEL sources. Ultrafast processes can be studied using double pulse schemes with sub-picosecond delays^113–115^, with further possibilities opened by those which offer significantly different photon energies. However, the DAC community is primarily interested in longer timescale dynamics (ps–ns) governing hydrodynamic expansion, heat transport, and structural transformations.

Later timescales can be accessed using two- (or multi-) bucket schemes^26^ which provide delay times corresponding to increments of the accelerator RF frequency (350 ps for LCLS and 770 ps for EuXFEL)—far shorter than the minimum spacing in the EuXFEL pulse train—and range to hundreds of nanoseconds. This scheme is currently in use at LCLS^95,116^, and is expected to be available at EuXFEL in 2027. In DAC experiments, two-bucket schemes will enable direct probing of the lattice evolution as samples cool via thermal conduction—capturing the earliest stages of melting and recrystallization, and constraining the thermodynamic pathway induced by XFEL heating. Such experiments will be invaluable in verifying FEA models, and will greatly aid the interpretation of results on planetary-relevant samples, i.e., to determine if high *T* metastable phases (e.g., bcc-Fe) form on quenching from the melt. Detection of individual pulses at sub-nanosecond delays is challenging as they will typically be collected on a single detector image. This makes probing the initial isochorically heated state challenging, as, in the absence of a phase transition, diffraction from the pump and probe pulses will be overlaid. Detectors capable of resolving X-ray pulses at nanosecond temporal separations are currently under development and will greatly extend what can be done with multi-bucket XFEL schemes^117^.

Hard X-ray split-and-delay lines (SDLs)^25,118–120^, in which pulses are split using Bragg crystal optics and recombined after a time delay is introduced in one of the paths, offer continuously tunable delays of up to several hundred picoseconds. SDLs can access hydrodynamic processes driven by thermal expansion to facilitate the release of thermal *P*, which may be especially complex for multi-layer sample assemblies (e.g., when samples are thermally insulated from the diamonds). SDLs also enable techniques such as transient-grating spectroscopy for direct measurements of heat diffusion and sound speeds in DACs^121^.

Beyond faster timescales, new opportunities will soon arise from the ability to shape the intensity envelope of EuXFEL pulse trains^122–124^. The sample *T* generally follows the pulse-energy distribution, resulting in rapid *T* stabilization for flat-top intensity profiles. Ramped profiles providing more gradual heating have been beneficial for capturing nucleation events in short-lived, metastable states^22^, whereas more complex profiles will enable thermal cycling within a single pulse train, e.g., back and forth across a phase line to access metastable pathways. Routes to shot-to-shot intensity control within pulse trains via variable bunch charge have already been implemented. Pulse intensity control within user experiments is subject to in-house commissioning, which will be undertaken within the next 12 months.

The strategic initiative at EuXFEL to provide access to hard X-rays above 30 keV^125,126^ will extend X-ray pump-probe DAC studies to higher photon energies. This relies on the ongoing development of high-frame-rate detectors with high-*Z* sensors^127^. These experiments will be greatly beneficial for total scattering measurements of disordered systems (e.g., liquids, amorphous materials), which require access to a wide range of *q*-space, as well as further reducing unwanted X-ray heating in isothermal compression experiments using the dDAC.

Overall, the coupling of DACs with exceptionally brilliant XFEL sources has opened new opportunities in high *P* science. Further advancements in this field will be driven by the wealth of new scientific opportunities offered by a combination of unexploited and forthcoming facility developments, including access to higher photon energies and advanced pulse modes, as well as the implementation of new X-ray diagnostics. These developments promise a more comprehensive understanding of the response of statically compressed samples to intense X-ray irradiation, which is essential to determine how experimental results can be related to planetary interiors, identify non-equilibrium processes triggered by X-ray irradiation, and extend rapid compression experiments to high *T*.
